# The effects of pre- and post-partum depression on child behavior and psychological development from birth to pre-school age: a protocol for a systematic review and meta-analysis

**DOI:** 10.1186/s13643-019-1267-2

**Published:** 2020-06-19

**Authors:** Lea Takács, Vít Kandrnal, Šárka Kaňková, František Bartoš, Jiří Mudrák

**Affiliations:** 1 Department of Psychology, Faculty of Arts, Charles University, Celetná 20, 116 42 Prague 1, Czech Republic; 2 Institute for Research on Children, Youth and Family, Faculty of Social Studies, Masaryk University, Joštova 10, 602 00 Brno, Czech Republic; 3 Department of Philosophy and History of Science, Faculty of Science, Charles University, Viničná 5, 128 44 Prague 2, Czech Republic

**Keywords:** Depression, Antenatal Depression, Postpartum Depression, Perinatal Depression, Pregnancy, Child Development, Child Health

## Abstract

**Background:**

Pre- and post-partum depression is a common mood disorder with detrimental effects on both mother and child. The aim of the proposed review is to summarize evidence related to the effects of both pre- and post-partum depression on child behavior and development from birth to preschool age. In particular, our review will address mutual relations between pre- and post-partum depression in order to determine whether pre- and post-partum depression predict child psychological outcomes independently, whether there is an effect of timing of depression on child outcomes, whether pre- and post-partum depression interact to affect child outcomes, and whether the effect of pre-partum depression is mediated by depression after child’s birth.

**Methods:**

We will include prospective longitudinal studies that report data about the effects of both pre- and post-partum depression on child psychological outcomes as published in peer-reviewed academic journals since January 1998. We will search EMBASE, MEDLINE, PsycARTICLES, PsycINFO, ISI Web of Science, Scopus, and Wiley Online databases to identify original research articles written in English. Two independent reviewers will screen search results in two stages: (i) titles and abstracts and (ii) full text. The first one will extract data into tables, while the latter will verify whether the data extracted are correct. We will assess the risk of bias in the selected studies using the Critical Appraisal Skills Programme (CASP), Cohort Study Checklist. The results of the review will be reported in a narrative form. If there are sufficient data available, a meta-analysis will be conducted using metaSEM package in R.

**Discussion:**

The proposed review will be the first systematic review summarizing the effects of both pre- and post-partum depression on child psychological development and behavior from birth to preschool age. The results of such a review may contribute to a better understanding of mutual relations between pre- and post-partum depression in their effects on child outcomes. They may also shed light on what periods in early human development are most vulnerable to the effects of maternal depression.

**Trial registration:**

PROSPERO CRD42018106269

## Background

Perinatal depression is a relatively frequent condition that affects between 5.2 and 13.0% of mothers in developed countries [1] and possibly up to 20% of mothers in low- and middle-income countries [2]. A recent meta-analysis estimates that the global prevalence of perinatal depression stands at 17.7% [3]. According to some authors, the figure would be even higher if screening was routinely applied in maternity health services [4].

The Diagnostic and Statistical Manual of Mental Disorders (DSM-5) [5] defines depression in perinatal period as a major depressive disorder with onset in the peripartum period up to 4 weeks after delivery, but according to some authors evidence supports also an expanded concept of childbirth-related depression which includes the entire first postpartum year [6–8]. Given the similarities between depressive symptoms and general symptoms accompanying pregnancy or postpartum period, detection of perinatal depression is a challenging task. Moreover, many women may feel reluctant to report negative emotions after the birth of their child and are more likely to report physical rather than psychological issues to their obstetricians [9]. These factors help account for the fact that postpartum depression is often unnoticed by healthcare professionals and remains, for the most part, underdiagnosed and untreated.

The factors commonly associated with perinatal depression include family history of depression, personal history of depressive and/or anxiety symptoms, overall poor health, low socioeconomic status, negative birth experience [10], and low partner support [11–13]. One of the strongest predictors of postpartum depression is antenatal depression [14, 15]. It is, however, unclear whether postpartum depression is a continuation of antenatal depression or rather an independent condition. In a recent review, antenatal depression has been reported as more prevalent than its postpartum counterpart [15], but the prevalence rates differ across the studies according to trimester, sampling, type of assessment used, and cut-off points applied [16]. Another review found similar prevalence rates for pre- and post-partum depression, with a somewhat higher upper limit for postpartum depression [6].

There is convincing evidence that maternal depression may have detrimental effects on child neurodevelopment and behavior [17–20]. Infants of depressed mothers show increased motor and cry response to unknown stimuli [21, 22] and lower social engagement [23] than children of non-depressed mothers. They also score higher on negative affectivity [24, 25] and more frequently develop insecure attachment with the mother [26]. The effect of maternal depression on the offspring seems to be long-lasting. For instance, Pawlby et al. [27] linked antenatal depression with increased risk of depression in adolescents.

The pathways through which depression may affect child development seem to be different for depression during pregnancy and depression in the postpartum period [20]. During pregnancy, maternal depression may affect placental function and via this route influence the programming of fetal neurodevelopment [28]. After birth, depression might affect child outcomes via changes in maternal bonding, parenting sensitivity and behaviors, and maternal self-esteem [23, 25, 29]. It is especially in the first years of their life that children are particularly vulnerable to the effects of maternal depression since these early years represent a period of rapid brain development and maturation of key physiological systems, such as HPA axis [30], leaving the developmental trajectories more open to external influences [31]. Apart from the mediating factors underlying the association between maternal depression and child developmental outcomes, existing studies identified several factors functioning as moderators, affecting the strength or direction of such association: children of mothers with higher socioeconomic status, education, or more optimal parenting behaviors have been found less likely to suffer from the adverse effects of maternal postpartum depression [20].

Despite a relatively high prevalence of pre-partum depression, multiple studies examined the effects of postpartum depression without paying attention to the potential effects of depression in the prenatal period while the studies on the effects of antenatal depression often failed to follow-up mother-child pairs in the postpartum period. Clearly enough, studies spanning both antenatal and postpartum period are needed to examine whether the effect of antenatal depression is mediated by postpartum depression and whether antenatal and postpartum depression have additive effects or interact to affect child behavior and development. Such studies are also needed to disentangle the effects of depression at different time periods and to determine the vulnerable phases or sensitive periods in child development, since exposure to maternal depression at different time periods may lead to different outcomes. Summarizing the current knowledge on the effects of perinatal mental disorders on child outcomes, Stein et al. [20] pointed out that there was some evidence indicating independent effects of pre- and post-partum depression on child outcomes and that the timing of depression might have different effects on different developmental domains. Yet, it is a matter of further research to know whether pre- and post-partum depression have additive, interactive, or cumulative effects.

Existing reviews accounting for both pre- and post-partum depression focused on infant health status [32] or infant growth [33]. Kingston and Tough [34] reviewed the articles that reported on the effects of maternal mental health problems in pregnancy or postpartum period on development in school-aged children. Although an increasing number of studies covers both antenatal and postpartum period to investigate the effects of maternal depression on child development, to the best of our knowledge, no systematic review has as yet examined the effects of both pre- and post-partum depression (with postpartum depression defined as spanning the first postpartum year) to determine their relative contribution to child developmental problems from birth to preschool age.

## Objectives

Our aim will be to review what is known about the effects of pre- and post-partum depression on child psychological development and behavior from birth to pre-school age (i.e., 5 years of age). We will only include studies reporting the effects of both pre- and post-partum depression. In particular, we will focus on the following questions:

1. Do pre- and post-partum depression predict child psychological outcomes independently?
2. Is there a sensitive period during which maternal depression is likely to have a higher impact on child psychological outcomes, i.e., is the effect of maternal depressive symptoms stronger in the prenatal or in the postnatal period?
3. Is the effect of pre-partum depression mediated by the effect of postpartum depression?
4. Is there an interaction between pre- and post-partum depression with respect to their impact on child psychological outcomes?

## Methods/design

Our intention is to conduct a systematic review of studies that investigate the effects of both pre- and post-partum depression on child behavior and psychological development from birth to the age of five. The review has been registered with PROSPERO (International prospective register of systematic reviews) at the National Institute for Health Research and the Centre for Reviews and Dissemination (CRD) at the University of York (registration no.: CRD42018106269). This protocol is reported according to the Preferred Reporting Items for Systematic Review and Meta-Analysis Protocols (PRISMA-P) [35] (see Additional file 1).

### Inclusion criteria

Studies will be included in the systematic review and meta-analysis based on the following criteria.

#### Study design

We will include empirical observational studies with a prospective longitudinal design. We will exclude studies with retrospective or cross-sectional designs, qualitative analyses, reviews, meta-analyses, and case studies. By restricting the review to prospective longitudinal studies, we intend to include studies reporting on maternal depression at given time points during pregnancy and postpartum period and not only retrospectively. Since our aim is to compare the effects of maternal depression at different time points, the studies with a longitudinal design should provide us with stronger indicators of causal relations than the studies with a cross-sectional design. Nevertheless, we are aware that causality cannot be proven based on solely observational data.

#### Participants

We will include mother-child pairs with children aged 0 to 5 years (pre-school age). This age restriction is necessitated by the large number of studies on the subject: we have decided to restrict the age range to make the review manageable. In addition, the first years of life represent a particularly vulnerable phase for brain development and maturation of the key physiologic systems. Maternal influence on child is highest in the first postpartum years; once the child starts attending primary school, there become many other factors involved that might affect child outcomes.

#### Exposure

We will assess the effects of maternal depression both during pregnancy (in one or more trimesters of pregnancy) and postpartum period. To achieve our objectives, we will only include studies that report data about both pre- and post-partum depression. As noted above, depressive disorder related to pregnancy and childbirth is defined in the DSM-5 as depression with peripartum onset up to 4 weeks after delivery. Nevertheless, we decided to include studies reporting on maternal depression up to 12 months after the child’s birth. Such decision seems justified as many authors recommend extending the concept of peripartum depression to the entire first postpartum year [6–8] given the high prevalence of depressive episodes occurring after the first postpartum month.

Moreover, we will include only those studies that assessed depression using cut-off scores on self-report validated scales (such as Edinburgh Postnatal Depression Scale (EPDS), Center for Epidemiologic Studies Depression Scale (CES-D), or Beck Depression Inventory (BDI)) indicating positive screening for pre- and post-partum depression, diagnostic interview, or hospital records. We will not include studies that assessed the effects of antidepressant medication on child behavior or development but only studies focusing on the effect of maternal depression.

#### Outcomes

Since various existing research shows that maternal depression may affect not only the child’s socioemotional but also cognitive and overall psychomotor development, we will not limit our investigation of child outcomes to the emotional domain. Our aim is to assess the impact of maternal depression on all of the following domains of child development: psychomotor, cognitive, socioemotional, and behavioral development, including temperament and behavioral difficulties. We will include only studies that examine psychological, not biological or biochemical outcomes in children (such as health condition, hormone levels, brain development). Our intention is to include not only studies that used objective measures (such as direct psychological assessment of child’s development) but also those which relied on maternal reports on child behavior and development, provided they were collected using validated tools. As noted above, we will include only studies which assess child behavior and development at a given time point and not retrospectively. Moreover, we will only include studies that focus on child-related and not maternal outcomes, so that for instance studies on child attachment patterns will be included, but studies on maternal bonding will not.

### Search methods

We will search the following electronic bibliographic databases: EMBASE, MEDLINE, Psycarticles, PsycINFO, ISI Web of Science, Scopus, and Wiley Online Library. Titles and abstracts only will be searched. We will restrict our search to studies written in English and published from 1 January 1998 in peer-reviewed academic journals. Moreover, apart from studies selected by systematic search of the databases listed above, we will also screen references listed in the selected studies to identify other sources that might be relevant to our review and add studies identified by a manual search. Prior to the final analysis, we will re-run our searches to make sure that recent studies meeting our selection criteria are also included.

The search strategy has been developed in cooperation with an experienced university librarian. The university librarian will run the search including deduplication of the results. A final search strategy is presented in Additional file 2.

### Selection of studies

A two-step selection process will be adopted. In the first step, titles and abstracts of studies identified by the search will be screened for eligibility independently by two team members using the following criteria:

1. Does the study report on maternal depression both during pregnancy and the postpartum period (up to 1 year postpartum)? Yes/No/Not clear
2. Does the study report on child psychological outcome/s (psychomotor development, cognitive development, social-emotional development, temperament, behavior)? Yes/No/Not clear
3. Is the age of children 0 to 5 years? Yes/No/Not clear
4. Does the study have a prospective longitudinal design? Yes/No/Not clear

All studies rated “Yes” or “Not clear” will be screened against the same criteria in the second step, where their full texts will be examined to determine whether they meet our criteria or not. Articles will be reviewed for suitability by two independent researchers. In case of disagreement, a third reviewer will make the final decision.

The eligibility criteria for the second stage of the selection process:

1. Does the study report on maternal depression both during pregnancy and the postpartum period (up to 1 year postpartum)? Yes/No
  - Does the study use a validated tool with a set cut-off to indicate positive screening for depression or a psychiatric interview to assess depression? Yes/No
2. Does the study report on child psychological outcome/s (psychomotor development, cognitive development, social-emotional development, temperament, behavior)? Yes/No
  - Does the study apply a validated tool/method to assess child outcomes? Yes/No
3. Is the age of children 0 to 5 years? Yes/No
4. Does the study have a prospective longitudinal design? Yes/No

Possible disagreement between reviewers at this stage of the selection process will be addressed in a team discussion, i.e., in a group including at least one other team member aside from the two reviewers who assessed the article in question.

### Risk of bias (quality assessment)

Methodological quality of the selected studies will be assessed using the Critical Appraisal Skills Programme [36]. This checklist consists of 12 questions covering cohort recruitment, exposure and outcome measurement, adjustment for confounding factors, follow-up measurement, reliability, and validity of study results. Quality assessment will be conducted by two research team members independently, and a third reviewer will be consulted in case of discrepancy.

### Data extraction

Data extracted from the selected studies should include title of the paper, authors’ names, year of publication, country where the study was conducted, year/s when the sample was recruited, method/s of participant recruitment, sample size, attrition rate, exclusion criteria, sample characteristics (maternal age, parity, marital status, socioeconomic status, ethnicity, delivery mode, birth weight, gestational age, newborn health status, child’s sex, breastfeeding, maternal health status in both pregnancy and postpartum period, smoking and alcohol consumption during pregnancy and postpartum period, a history of depression), depression assessment in pregnancy and postpartum (timing, measure used for assessing depression, cut-off used for identifying women with a positive screening for depression, depression prevalence, type of informant: self-reported/objective assessment), offspring assessment/s (timing, measure/s, type of informant), confounding variables included, quantitative results on association between pre-/post-partum depression and child outcomes (correlation coefficients, odds ratios, beta coefficients, SE, confidence intervals, *p* values), and information about possible moderators/mediators. The data will be extracted by two research team members. The first one will extract data into tables, while the latter will verify whether the data extracted are correct.

### Strategy for data synthesis

Our intention is to present the findings in the form of a narrative synthesis. We will summarize the results of the selected studies relevant for our four main review questions: the independent effects of pre- and post-partum depression on child outcomes, the vulnerable phase for the effects of depression on the child (whether the effect is more detrimental when child is exposed to depression in the prenatal or in the postpartum period), possible mediation of the effect of pre-partum depression by postpartum depression, and possible interaction between pre- and post-partum depression in their effects on child outcomes. In addition, we aim to summarize the results of the selected studies as regards the potential covariates, moderators, and mediators of the association between pre- and post-partum depression and child outcomes.

The following information will be presented in tables accompanying the narrative synthesis: author, year of publication, country where the study was conducted, sample size, specific sample characteristics (primiparous/multiparous women, medically low-/high-risk women, low-/high-income families etc.), timing of depression measurement in pregnancy (gestational week), timing of depression measurement in the postpartum period (weeks/months), instrument used to measure pre- and post-partum depression and a cut-off set to identify women with a positive screening for depression, prevalence of pre- and post-partum depression (positive screening for depression or psychiatric diagnosis), child outcomes, an instrument used to measure child outcomes and type of informant, timing of measurement of child outcomes, confounders included, association between pre- and post-partum depression and child outcomes, moderating/mediating role of postpartum depression, and other moderators/mediators. The individual tables will be divided into several parts by child outcome (we plan to develop separate tables for particular areas of child development).

The selected studies will be examined for their potential to be included in a meta-analysis. If possible, we will examine the review objectives 1, 2, and 3 by means of the meta-analysis: we will examine the independent contribution of pre- and post-partum depression to child outcomes, compare their effects size to assess the effect of timing of depression, and analyze whether the effect of prenatal depression on child outcomes is mediated by postpartum depression. We also intend to investigate the differences in child outcomes according to various types of measurement of maternal depression (various questionnaires to assess depression; maternal report/objective assessment) and the effects of maternal depression on various child outcomes (e.g., different domains of development) and on the same child outcomes measured at different developmental stages (infants, toddlers, preschoolers). If there are sufficient data available, we will also report the results for specific subgroups (primiparae vs. multiparae, groups with different socioeconomic status, medically low-risk vs. medically high-risk women etc.).

Where appropriate, we will conduct a separate meta-analysis for each type of outcome using a meta-analytic structural equation modeling (MASEM) framework [37]. We will follow the recommendation outlined by Sheng et al. [38] and utilize a two-stage MASEM, which estimates a pooled meta-analytic correlation matrix in the first stage that is subsequently used to fit a structural equation model in the second stage [39]. The independence of pre- and post-partum depression will be assessed as a direct effect while the mediation of the pre-partum depression effect by post-partum depression will be assessed as an indirect effect in the mediation MASEM framework, with the combined effect of pre- and post-partum depression assessed as a total effect.

For the analysis, we will extract bivariate correlation coefficients between the pre- and post-partum depression and the dependent variable. In order to ensure that the elements in the correlation matrices are generated in the similar way, we will only include studies identified on the base of our inclusion criteria. We will use the random-effect version of the two-stage MASEM which allows to account for the missing correlations between the variables of interest. Following the first stage, the heterogeneity of the estimated meta-analytic correlation matrix will be tested using a Q-test and evaluated by *I*^2^ statistics. In case of heterogeneity, we will assess its impact by re-estimating the MASEM for different subgroups of possible moderators mentioned above (e.g., different methods for measuring depression) and by comparing the resulting models [40–42]. The results will be interpreted with a focus on effect sizes. The meta-analysis will be conducted in R [43] using the metaSEM package [44].

### Dissemination

A manuscript containing the review results will be submitted to an international peer-reviewed journal. The findings will also be presented at a scientific conference. If it turns out that our findings could be of interest to the public, we will disseminate them through the mass media.

## Discussion

The aim of this systematic review is to provide a summary of existing knowledge regarding the effects of pre- and post-partum depression on child behavior and development during a period spanning from birth to the age of five. To the best of our knowledge, this review will be the first to use only studies that report on both pre- and post-partum depression to compare the effects of both conditions on child behavior and development.

Maternal depression is one of the most frequent complications related to childbirth, but little is known about which are the most sensitive stages in the early development, i.e., at what stage of fetal/infant development is exposure to maternal depression most likely to affect developmental outcomes. Moreover, the relation between pre- and post-partum depression and their distinct or combined effects on child remain unclear. The results of this review may help determine the phases of early development that are most vulnerable to maternal depression. This may be helpful for clinicians to identify children who are at high risk of adverse outcomes and could benefit from timely intervention.

## Supplementary information

**Additional file 1.** The Preferred Reporting Items for Systematic Reviews and Meta-Analyses for Protocols (PRISMA-P) checklist was used in this protocol.

**Additional file 2.** Search strategy.

## Data Availability

Not applicable.

## Abbreviations

BDI: Beck Depression Inventory
EPDS: Edinburgh Postnatal Depression Scale
CES-D: Center for Epidemiologic Studies Depression Scale
