# Supplementary material for: The effects of pre- and post-partum depression on child behavior and psychological development from birth to pre-school age: a protocol for a systematic review and meta-analysis
Source: Syst Rev. 2020 Jun 19;9:146. doi: 10.1186/s13643-019-1267-2 (PMC7304193; doi:10.1186/s13643-019-1267-2)
Supplement: Supplementary file 2 — Additional file 2. Search strategy. [file 13643_2019_1267_MOESM2_ESM.docx]

**The effects of pre- and post-partum depression on child behavior and psychological development from birth to pre-school age: A protocol for a systematic review and meta-analysis**

| **Database** | **Search syntax** |
| --- | --- |
| EMBASE | ((antenat* or antepart* or prepart* or prenatal* or pregnan* or perinatal*) and (postnat* or postpart* or perinatal*) and depress* and (child* or infant* or offspring*) and (development* or behavio*r or temperament* or function* or outcome* or difficult* or disturb*)).ab. |
| Medline | AB ( (antenat* or antepart* or prepart* or prenatal* or pregnan* or perinatal*) ) AND AB ( (postnat* or postpart* or perinatal*) ) AND AB (depress*) AND AB ( (child* or infant* or offspring*) ) AND AB ( (development* or behavio*r or temperament* or function* or outcome* or difficult* or disturb*) ) |
| Psycarticles | AB ( (antenat* or antepart* or prepart* or prenatal* or pregnan* or perinatal*) ) AND AB ( (postnat* or postpart* or perinatal*) ) AND AB (depress*) AND AB ( (child* or infant* or offspring*) ) AND AB ( (development* or behavio*r or temperament* or function* or outcome* or difficult* or disturb*) ) |
| Psycinfo | AB ( (antenat* or antepart* or prepart* or prenatal* or pregnan* or perinatal*) ) AND AB ( (postnat* or postpart* or perinatal*) ) AND AB (depress*) AND AB ( (child* or infant* or offspring*) ) AND AB ( (development* or behavio*r or temperament* or function* or outcome* or difficult* or disturb*) ) |
| ISI Web of Science | TS=( (antenat* or antepart* or prepart* or prenatal* or pregnan* or perinatal*) AND (postnat* or postpart* or perinatal*) AND (depress*) AND (child* or infant* or offspring*) AND (development* or behavio$r or temperament* or function* or outcome* or difficult* or disturb*) ) |
| SCOPUS | TITLE-ABS-KEY ( ( antenat* OR antepart* OR prepart* OR prenatal* OR pregnan* OR perinatal* ) AND ( postnat* OR postpart* OR perinatal* ) AND ( depress* ) AND ( child* OR infant* OR offspring* ) AND ( development* OR behavio#r OR temperament* OR function* OR outcome* OR difficult* OR disturb* ) ) |
| Wiley Online Library | (antenat* OR antepart* OR prepart* OR prenatal* OR pregnan* OR perinatal*) in Abstract AND (postnat* OR postpart* OR perinatal*) in Abstract AND (depress*) in Abstract AND (child* OR infant* OR offspring*) in Abstract AND (development* OR behavio*r OR temperament* OR function* OR outcome* OR difficult* OR disturb*) in Abstract |
